# The Impact of Cerebral Microbleeds Presence on Outcome Following Minor Stroke Treated With Antiplatelet Therapy

**DOI:** 10.3389/fneur.2020.00522

**Published:** 2020-06-16

**Authors:** Kenichi Sakuta, Hiroshi Yaguchi, Takeo Sato, Teppei Komatsu, Kenichiro Sakai, Hidetaka Mitsumura, Satoshi Matsushima, Yasuyuki Iguchi

**Affiliations:** ^1^Department of Neurology, Kashiwa Hospital, The Jikei University School of Medicine, Chiba, Japan; ^2^Department of Neurology, The Jikei University School of Medicine, Tokyo, Japan; ^3^Department of Radiology, The Jikei University School of Medicine, Tokyo, Japan

**Keywords:** cerebral microbleeds, ischemic stroke, prognosis, magnetic resonance image, susceptibility-weighted imaging

## Abstract

**Background and Purpose:** The relationship between cerebral microbleeds (CMBs) and prognosis in patients with ischemic stroke is still unclear. Our aim here was to verify the relationship between CMBs and functional outcomes in patients with minor ischemic stroke treated with antiplatelet therapy.

**Methods:** We retrospectively reviewed consecutive patients with a non-cardiogenic minor ischemic stroke (NIHSS <4 on admission) who underwent initial brain magnetic resonance imaging within the first 48 h following symptom onset. The patients were divided into two groups based on the presence or absence of CMBs and the two groups were adjusted using the pre-stroke modified Rankin scale (mRS). Poor outcome was defined as an mRS score in the 3–6 range measured 90 days after symptom onset. Logistic regression analyses were performed to determine the factors independently associated with poor outcome.

**Results:** A total of 240 patients (187 men, median age 66 years old) were enrolled in our study. There was a non-significant trend toward a worsening shift of 3-month mRS score distribution in the CMB group compared with the no-CMB group. Multivariate analysis revealed that the presence of CMBs was independently predictive of poor outcome (OR, 3.44; 95% CI, 1.08–10.93; *P* = 0.036).

**Conclusion:** Our findings suggest that the presence of CMBs is the predicting factor of poor outcome in minor ischemic stroke patients.

## Introduction

Cerebral microbleeds (CMBs) are considered biomarkers of cerebrovascular disease, and more specifically of small vessel disease (1, 2). Stroke patients with CMBs have higher recurrence rates for both ischemic and hemorrhagic strokes (3–5), and have increased mortality rates (6–8) compared with stroke patients without CMBs. However, although the association between CMBs and stroke recurrence and/or mortality is well-established, little is known about the functional outcomes of stroke patients with CMBs. The aim of the present study was to investigate whether CMBs affect the functional outcome of patients presenting with minor ischemic stroke.

## Methods

### Study Design

This retrospective study included consecutive ischemic stroke patients who were admitted between October 2012 and June 2018 to Jikei University Hospital, Japan. To exclude the possible effects of antithrombotic agents, only ischemic stroke patients who were treated with antiplatelet agents were enrolled (9). Patients were eligible for this study if the admission occurred within 48 h after symptom onset and if they received a diagnosis of non-cardiogenic minor stroke according to the TOAST classification (10), with baseline National Institutes of Health Stroke Scale (NIHSS) <4. Based on our institutional protocol, we performed the following examinations to determine the stroke subtype: electrocardiogram monitoring for at least 7 days, echocardiogram, carotid artery ultrasonography, transcranial color Doppler, and transesophageal echocardiogram tests to investigate possible right-to-left shunt and aortic arch lesions. We additionally performed a brain magnetic resonance imaging (MRI) scan on admission and a second scan 7 days after symptom onset. CMBs were identified and documented using susceptibility-weighted imaging (SWI). Our institutional protocol for treating non-cardiogenic stroke patients was as follows: single antiplatelet therapy for patients enrolled between October 2012 to April 2017, and dual antiplatelet therapy for patients enrolled between May 2017 to June 2018. The dual antiplatelet therapy was prescribed for approximately 2 weeks and was followed by the use of a single antiplatelet agent. Combining edaravone depended on the treating physician's assessment. Participant exclusion criteria were as follows: (1) treatment with recombinant tissue plasminogen activator (rt-PA) and/or endovascular therapy; (2) treatment with anticoagulant agents for a cardioembolic, cryptogenic, or aortogenic stroke; (3) MRI counterindications; (4) premorbid modified Rankin scale (mRS) score of 3, 4, or 5; and (5) cases of stroke mimic. No participants were prescribed oral anticoagulant agents.

This study was approved by the Ethics Committee of the Jikei University School of Medicine [No. 29-195[8811]]. All study protocols and procedures were conducted in accordance with the declaration of Helsinki.

### Clinical Background

We collected the following clinical data from all patients: age, sex, time from symptom onset to admission, length of hospital stay, and traditional cardiovascular risk factors including: hypertension, dyslipidemia, diabetes mellitus, malignant neoplasms, and chronic kidney disease. The NIHSS was used on admission by a trained neurologist to quantify stroke severity. Ischemic stroke recurrence was defined as a new ischemic lesion evidenced by a brain MRI diffusion-weighted image (DWI) during hospitalization. A hemorrhagic complication was defined as the combination of intracranial hemorrhage and clinically overt systemic bleeding during hospitalization. The mRS was used to assess functional recovery 90 days after stroke onset via an outpatient clinic or a telephone interview, and a score within the 3–6 range was defined as a poor outcome. These assessments were performed by trained neurologists, the majority of whom were physicians-in-charge.

### Imaging Acquisition

The parameters for brain imaging are included in the Supplementary Methods. A CMB was defined as a round, focal, low-intensity lesion within the parenchyma that measured up to 10 mm in size as viewed using SWI, according to a recent consensus (2, 11). Symmetrical subcortical and hypointense lesions, indicating focal calcification, were excluded. Low-intensity lesions within an infarct were considered to be hemorrhagic transformations rather than CMBs. The locations of CMBs were classified based on a previous report (12), as follows. Patients with CMBs in the basal ganglia, thalamus, or infratentorial region (deep locations), but without lobar CMBs, were defined as hypertensive (HTN) types. Patients with strictly lobar CMBs were defined as cerebral amyloid angiopathy (CAA) types. Patients with a combination of mixed lobar and deep CMBs were defined as mixed types. Two neurologists (KS and HM), who were blinded with respect to the clinical information associated with the SWI images, independently evaluated the SWI images. In cases of initial disagreement, the final number was reached through consensus. The κ statistic of agreement between the two neurologists was 0.84. To evaluate the relationship between CMBs and deep white matter hyperintensities (DWMHs), the two neurologists also evaluated DWMHs in fluid-attenuated inversion recovery (FLAIR) images using the Fazekas rating scale (13). The κ statistic of agreement for DWMHs was 0.88.

### Statistical Analyses

To minimize intergroup differences, we adjusted for pre-stroke mRS scores. The patients were divided into two groups: patients with CMBs (CMB group) and patients without CMBs (no-CMB group). Continuous and categorical variables are described with medians (interquartile range [IQR]) and counts (%), respectively. First, we compared the patient characteristics, clinical backgrounds, laboratory findings, choice of treatment, and clinical outcomes between groups using the chi-squared test and Fisher exact test for categorical variables, and the Mann–Whitney U test for continuous variables. Systolic blood pressure on admission was the only factor to meet the criteria for normal distribution (*P* = 0.051), and a *t*-test was used. A corresponding *P*-value <0.05 was considered as statistically significant. Second, we performed a multivariate logistic regression analysis to extract factors that were independently associated with the presence of CMBs. Variables with *P*-value <0.200 in the univariate analysis were then entered into the multivariate analysis. Third, the distribution of 3-month mRS scores in each group was compared using a Kruskal–Wallis test, because it did not have a normal distribution (*P* < 0.001). Fourth, we performed a second and third multivariate logistic regression analysis to evaluate the factors that were independently associated with stroke recurrence and hemorrhagic complication. The multivariate analysis for ischemic stroke recurrence included well-known risk factors for CMBs: age, history of diabetes mellitus and stroke, and a stroke subtype of large artery atherosclerosis (LAA) (14–16). The multivariate analysis for hemorrhagic complication included well-known risk factors for CMBs: history of stroke, a stroke subtype of small vessel occlusion (SVO), and dual-antiplatelet therapy (17–19). Fifth, we compared the outcome differences between the CMB location subgroups (HTN, CAA, and mixed) using a Kruskal–Wallis test. Finally, we performed a fourth multivariate logistic regression analysis to determine the factors that were independently associated with poor outcome. The multivariate analysis included well-known risk factors for poor outcome: age, NIHSS on admission, LAA, DWMHs, and ischemic stroke recurrence (16, 20–25). All statistical analyses were performed using the SPSS (v22 for Windows; SPSS Inc., Chicago, IL, USA) statistical software package.

## Results

### Patients

**Figure 1:**
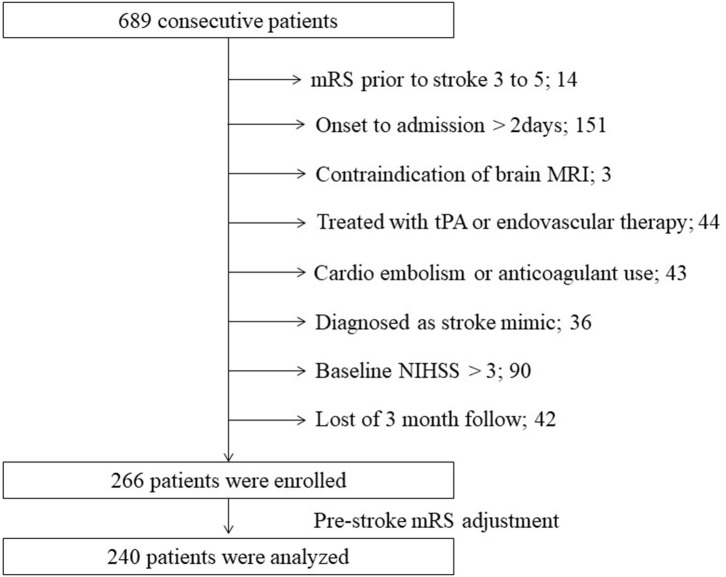
Flow diagram of patient selection. MRI, magnetic resonance imaging; mRS, modified Rankin scale; NIHSS, National Institutes of Health Stroke Scale; tPA, tissue plasminogen activator.

**Figure 2:**
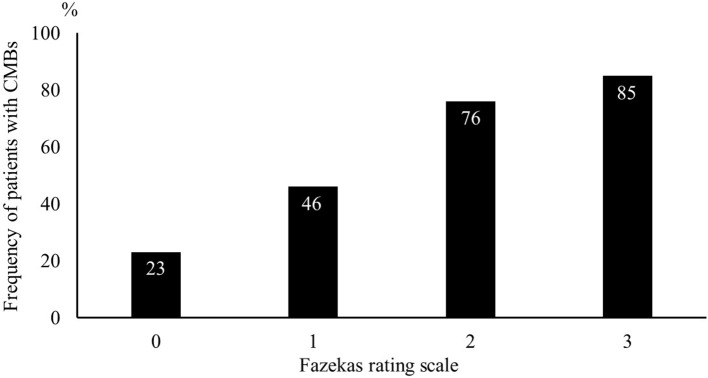
Frequency of patients with cerebral microbleeds using the Fazekas scale. The likelihood of cerebral microbleeds being present increased gradually with higher Fazekas scale scores.

Of the 689 consecutive patients admitted during the study period with an ischemic stroke, 266 were enrolled in our study (Figure 1). After adjustment for pre-stroke mRS, the two groups had 120 patients each. Clinical characteristics of the study population are shown in Table 1. The median age was 66 years, and 187 patients (78%) were men. The median NIHSS on admission was 1 (IQR 1–2). All patients received antiplatelet therapy, of which 90 (38%) were prescribed dual antiplatelet therapy. Figure 2 shows that the frequency of patients with CMBs was positively correlated with the Fazekas scale in a linear fashion (*P* < 0.001).

**Table 1:** Baseline characteristics of the study population.

		**Microbleeds**	
	**Total (*n =* 240)**	**CMB (*n =* 120)**	**No-CMB (*n =* 120)**	***P***
Men, n (%)	187 (78)	92 (77)	95 (79)	0.641
age, median (IQR)	66 (57–76)	68 (61–80)	62 (53–70)	<0.001
Medical history				
Diabetes Mellitus, n (%)	74 (31)	33 (28)	41 (34)	0.263
Hypertension, n (%)	165 (69)	90 (75)	75 (63)	0.037
Dyslipidemia, n (%)	126 (53)	58 (48)	68 (57)	0.196
Ischemic heart disease, n (%)	20 (8)	14 (12)	6 (5)	0.062
Peripheral arterial disease, n (%)	6 (3)	4 (3)	2 (2)	0.342
Stroke, n (%)	43 (18)	29 (24)	14 (12)	0.012
Chronic kidney disease, n (%)	33 (14)	19 (16)	14 (12)	0.349
Malignant neoplasms, n (%)	17 (7)	9 (8)	8 (7)	0.801
Physical examination				
Body Mass Index, median (IQR)	23.7 (21.7–26.0)	23.6 (21.8–25.8)	23.9 (21.6–26.1)	0.327
systolic blood pressure, mmHg, median (IQR)	163 (140–183)	171 (147–186)	154 (138–179)	0.065
heart rate, /minute, median (IQR)	79 (70–90)	80 (72–90)	76 (68–88)	0.344
NIHSS, median (IQR)	1 (1–2)	1 (1–2)	1 (0–2)	
NIHSS, average (SD)	1.4 (1.0)	1.5 (1.0)	1.3 (1.0)	0.087
Laboratories				
Glucose, mg/dL, median (IQR)	114 (101–143)	115 (99–146)	113 (104–135)	0.933
eGFR, mL/min/1.73 m^2^, median (IQR)	74.4 (55.7–98.7)	67.7 (53.1–88.5)	85.8 (63.2–103.7)	0.001
BNP, pg/mL, median (IQR)	20.7 (11.1–41.8)	26.3 (14.0–57.2)	15.9 (8.8–34.1)	0.001
TOAST classification				
Large Artery Atherosclerosis, n (%)	32 (13)	15 (13)	17 (14)	0.704
Small vessel occlusion, n (%)	92 (38)	60 (50)	32 (27)	<0.001
Other determined etiology, n(%)	43 (18)	16 (13)	27 (23)	0.064
Undetermined etiology, n(%)	73 (30)	29 (24)	44 (37)	0.035
Treatment				
Single antiplatelet agent, n (%)	150 (63)	73 (61)	77 (64)	0.594
Dual antiplatelet agents, n (%)	90 (38)	47 (39)	43 (36)	0.594
Edaravone combined, n (%)	94 (39)	42 (35)	52 (43)	0.186
Hospital stay, day, median (IQR)	12 (9–16)	12 (9–18)	11 (9–15)	0.162
Hemorrhagic complication, n(%)	6 (3)	4 (3)	2 (2)	0.342
Systemic bleeding, n (%)	5 (2)	3 (3)	2 (2)	0.500
Intracranial hemorrhage, n (%)	1 (0.4)	1 (0.8)	0 (0)	0.500
Ischemic stroke recurrence, n (%)	23 (10)	13 (11)	10 (8)	0.511

*BNP, brain natriuretic peptide; eGFR, estimated glomerular filtration rate; IQR, interquartile range; NIHSS, national institutes of health stroke scale*.

### Comparison Between Patients With and Without CMBs

**Figure 3:**
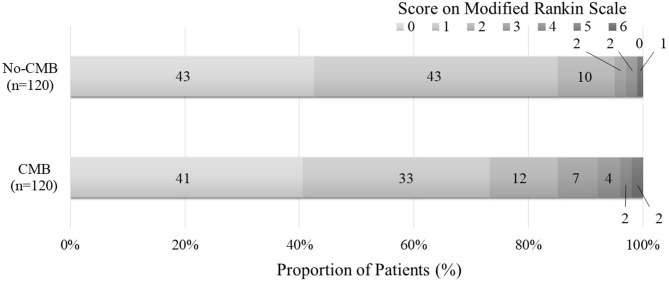
Functional outcomes at 90 days as a function of modified Rankin scale scores. The percentages of patients with scores from 0 to 6 on the modified Rankin scale (mRS) in the CMB and no-CMB groups are shown as follows: 0, no symptoms; 1, no clinically significant disability; 2, slight disability (able to handle own affairs without assistance but unable to carry out all previous activities); 3, moderate disability (requiring some help but able to walk unassisted); 4, moderately severe disability (unable to attend bodily needs and unable to walk); 5, severe disability (requiring constant nursing care and attention); and 6, death. In the no-CMB group, no patient had a score of 5. In our analysis, there was no significant difference between the CMB group and the no-CMB group in the overall distribution of scores (*P* = 0.195).

Compared with the no-CMB group, the CMB group patients had an older average age, higher frequency of a medical history of hypertension and stroke, lower estimated glomerular filtration rate, higher brain natriuretic peptide levels, higher frequency of SVO stroke subtype, and lower frequency of Undetermined etiology stroke subtype using the TOAST classification (Table 1). A trend toward a higher NIHSS on admission was observed in the CMB group. There was a positive correlation between SVO stroke subtype and DWMH, and a negative correlation between history of dyslipidemia and the presence of CMBs in the multivariate logistic regression analysis (Table 2). The frequency of antiplatelet prescription did not differ between the two groups (Table 1). The frequency of hemorrhagic complication appeared to be higher in the CMB group compared with the no-CMB group, but was not significantly different (3 vs. 2%, *P* = 0.342). Ischemic stroke recurrence also appeared to be more frequent in the CMB group, but again, there was no significant difference between the two groups (11 vs. 8%, *P* = 0.511). The frequency of poor outcomes (mRS 3–6) was significantly higher in the CMB group [*n* = 17 [14%] vs. *n* = 5 [4%], *P* = 0.007]. There was a trend toward a worsening shift of 3-month mRS scores in the CMB group compared with the no-CMB group, but there was no significant difference in the overall distribution of scores (*P* = 0.195, Figure 3).

**Table 2:** Univariate and multivariate logistic regression analyses for the presence of cerebral microbleeds.

	**Univariate**			**Multivariate**		
	***OR***	**95%CI**	***P***	***OR***	**95%CI**	***P***
Age	1.04	1.02–1.07	<0.001	1.03	0.99–1.07	0.103
Systolic blood pressure	1.01	1.00–1.02	0.067	1.01	1.00–1.02	0.050
History of stroke	2.41	1.20–4.84	0.013	1.91	0.78–4.71	0.158
History of hypertension	1.80	1.03–3.13	0.038	1.29	0.64–2.61	0.480
History of dyslipidemia	0.72	0.43–1.19	0.197	0.47	0.25–0.90	0.022
History of IHD	2.51	0.93–6.77	0.069	1.62	0.48–5.46	0.436
DWMH	2.82	2.05–3.88	<0.001	2.28	1.62–3.21	<0.001
Brain natriuretic peptide	1.01	1.00–1.01	0.034	1.00	1.00–1.01	0.220
eGFR	0.98	0.98–0.99	0.001	1.00	0.99–1.02	0.793
TOAST; SVO	2.75	1.60–4.72	<0.001	2.99	1.07–8.37	0.037
TOAST; Other determined etiology	0.530	0.27–1.05	0.067	1.24	0.40–3.82	0.712
TOAST; Undetermined etiology	0.550	0.32–0.96	0.036	1.078	0.38–3.08	0.890

*Variables with P <0.200 in the univariate analysis were entered into a multivariate logistic regression model to identify the variables independently associated with the presence of cerebral microbleeds*.

*CI, confidence interval; DWMH, deep white matter hyperintensity; eGFR, estimated glomerular filtration rate; IHD, ischemic heart disease; OR, odds ratio; SVO, small vessel occlusion; TOAST, Trial of Org 10,172 in Acute Stroke Treatment*.

### Factors Associated With Ischemic Stroke Recurrence and Hemorrhagic Complications

Multivariate logistic analyses for ischemic stroke recurrence and hemorrhagic complication are shown in Tables 3, 4. LAA was independently associated with ischemic stroke recurrence, and no factors were associated with hemorrhagic complication.

**Table 3:** Univariate and multivariate logistic regression analyses for ischemic stroke recurrence.

	**Univariate**			**Multivariate**		
	***OR***	**95%CI**	***P***	***OR***	**95%CI**	***P***
Age	1.01	0.98–1.05	0.579	0.99	0.96–1.03	0.762
History of diabetes mellitus	1.50	0.62–3.65	0.367	1.47	0.57–3.79	0.423
History of stroke	2.77	1.09–7.04	0.032	1.64	0.58–4.64	0.354
Large artery atherosclerosis	6.82	2.68–17.37	<0.001	6.26	2.29–17.13	<0.001
Cerebral microbleeds	1.34	0.56–3.18	0.512	1.42	0.54–3.77	0.480

*Well-known risk factors for ischemic stroke recurrence, such as age, history of stroke and diabetes mellitus, and large artery atherosclerosis stroke subtype, were entered into a multivariate logistic regression model with the presence of cerebral microbleeds to identify the variables independently associated with ischemic stroke recurrence*.

*CI, confidence interval; OR, odds ratio*.

**Table 4:** Univariate and multivariate logistic regression analyses for hemorrhagic complications.

	**Univariate**			**Multivariate**		
	***OR***	**95%CI**	***P***	***OR***	**95%CI**	***P***
History of stroke	2.35	0.42–13.28	0.332	1.87	0.31–11.10	0.493
Small vessel occlusion	0.31	0.04–2.73	0.294	0.25	0.03–2.30	0.223
Dual antiplatelet therapy	1.69	0.33–8.56	0.526	1.66	0.32–8.56	0.547
Cerebral microbleeds	2.03	0.37–11.32	0.417	2.39	0.40–14.23	0.338

*Well-known risk factors for hemorrhagic complications, such as history of stroke, small vessel occlusion stroke subtype, and dual antiplatelet therapy, were entered into a multivariate logistic regression model with the presence of cerebral microbleeds to identify the variables independently associated with hemorrhagic complications*.

*CI, confidence interval; OR, odds ratio*.

### CMBs Locations and Outcomes

Based on CMB locations, the patients were divided into three types: HTN type [*n* = 42 [35%]], CAA type [*n* = 28 [23%]], and mixed type [*n* = 50 [42%]]. The frequencies of poor outcomes (mRS 3–6) in these three types of patients were 12% (5/42), 14% (4/28), and 16% (8/50), respectively, and there were no significant differences between the three groups (*P* = 0.855).

### Factors Associated With Poor Outcome

Previously reported factors predicting a poor prognosis, such as age, NIHSS on admission, ischemic stroke recurrence, LAA stroke subtype, and DWMH, were analyzed using a univariate logistic regression analysis. Age, NIHSS, and LAA stroke subtype were significantly associated with poor outcome (mRS 3–6) in the present cohort. These factors were all subsequently entered into a multivariate logistic regression analysis, and the presence of CMBs was one of the factors that was independently associated with a poor outcome (OR, 3.44; 95% CI, 1.08–10.93; *P* = 0.036, Table 5).

**Table 5:** Univariate and multivariate logistic regression analyses for poor outcome.

	**Univariate**			**Multivariate**		
	***OR***	**95%CI**	***P***	***OR***	**95%CI**	***P***
Age	1.07	1.02–1.11	0.002	1.05	1.01–1.10	0.027
NIHSS on admission	1.69	1.07–2.66	0.024	1.69	1.06–2.70	0.029
Ischemic stroke recurrence	2.33	0.71–7.58	0.161	1.50	0.36–6.25	0.576
Large artery atherosclerosis	3.60	1.34–9.69	0.011	3.17	0.99–10.12	0.051
DWMH	1.27	0.87–1.86	0.217	0.88	0.55–1.40	0.592
Cerebral microbleeds	3.80	1.35–10.65	0.011	3.44	1.08–10.93	0.036

*Well-known risk factors for poor outcome, such as age, NIHSS on admission, ischemic stroke recurrence, large artery atherosclerosis stroke subtype, and DWMH, were entered into a multivariate logistic regression model with the presence of cerebral microbleeds to identify the variables independently associated with poor outcome (mRS 3–6)*.

*CI, confidence interval; DWMH, deep white matter hyperintensity; OR, odds ratio*.

## Discussion

The present study showed that the presence of CMBs was independently associated with a poor functional outcome in patients with minor stroke. The novelty of this study is that the association between CMBs and poor functional outcome in stroke patients was examined in great detail and was shown to be significant.

In the present cohort, there was a strong relationship between the presence of CMBs and a poor outcome, and a non-significant relationship between the presence of CMBs and ischemic stroke recurrence in patients with a minor stroke. It is generally agreed that a relationship between CMBs and subsequent intracerebral hemorrhage risk exists in stroke patients (3–5). Furthermore, the frequency of ischemic stroke recurrence, especially when severely disabling or fatal, is reported to be increased in stroke patients with CMBs (4, 6). Moreover, CMBs in stroke patients are reported to be strongly related to the likelihood of an in-hospital death (7). The present study is partially in line with these previous reports with regards to the poor prognosis of stroke patients with CMBs. The linking factor between the two is believed to be either the high frequency of stroke recurrence in CMB patients or the mechanisms by which CMBs are formed.

The CMB patients in our cohort had an approximately 1.5 times higher recurrence rate of ischemic stroke compared to those without CMBs, but this result was not statistically significant (11 vs. 8%, *P* = 0.511). A high frequency of recurrence contributes to poor prognosis in stroke patients (16). A previously published systematic review revealed that antithrombotic agents were significantly associated with intracerebral hemorrhage risk in stroke or transient ischemic attack patients with CMBs who were followed for a least a year (26). Consequently, physicians tend to limit the amount or the duration of antithrombotic drug use. Because the present study evaluated only the initial prescription of antiplatelet agents, the subsequent treatment strategies that were used may have also affected the results. Furthermore, because the occurrence of hemorrhagic stroke and ischemic stroke recurrence was evaluated over a very short period, during a median 12 days of hospitalization, the low frequency of hemorrhagic events or the non-significant difference in ischemic stroke recurrence may have been strongly affected by study design. However, although we should take these concerns into consideration, ischemic stroke recurrence was not an independent factor for poor prognosis in our multivariate analysis. In our cohort, stroke recurrence seemed to have only a weak impact as a factor for prognosis in patients with CMBs.

The mechanisms underlying CMBs may explain the relationship between CMBs and poor prognosis as follows. The presence and amount of CMBs or white matter hyperintensities (WMHs) are considered to be a surrogate marker of small vessel disease severity (27, 28). Small vessel disease reflects endothelial dysfunction, which leads to cerebral blood flow dysregulation, blood–brain barrier dysfunction, and endothelial inactivation (29, 30). The CMBs and WMHs are assumed to form as a result of these consequences of endothelial dysfunction. We speculate that the poor prognosis in patients with CMBs in the present study was caused by these mechanisms, because the degree of neural network damage should be affected by endothelial dysfunction (28). Recent studies have reported that a high prevalence of WMHs in ischemic stroke patients is strongly related to the poor recovery of neurological symptoms, functional impairment, and mortality (25, 31–33). With our results demonstrating a positive relationship between DWMH burden and the frequency of CMBs, the presence of CMBs in acute ischemic stroke patients may also contribute to poor functional recovery.

There are several limitations to our study. First, as mentioned previously in this discussion, the evaluation of subsequent hemorrhagic stroke and ischemic stroke recurrence was only performed over a short period, and this may have led to a low incidence being recorded. Second, the tracking period in this cohort was only 3 months. Furthermore, the use of telephone interviews may have over- or under-estimated the symptomology. Third, all patients in this study were Japanese, limiting the generalizability to other populations. In addition, the proportion of men in this cohort was relatively high. Although women are reported to have a lower stroke risk than men, there might have been a selection bias in our study (34). Fourth, the prescribed antiplatelet agents were heterogeneous. Although there were prescription protocols in place, it is still possible that physicians may have deviated from them. Fifth, the sensitivity for detecting CMBs is higher with 3T scanners than with 1.5T scanners, and because of the standard protocols in our facility, the slice thicknesses used were different (1.5 mm for 3T and 2 mm for 1.5T). These differences may have affected whether or not CMBs were detected. Sixth, 6% (42) of patients were lost at the 3-month follow-up. Finally, we performed this retrospective cohort study in a single center. However, this limitation can also be viewed as a strength because patients were able to be diagnosed with a non-cardiogenic stroke with a high degree of precision, using strict inspection standards. Future studies should investigate the choice of long-term antiplatelet treatment strategy on the functional outcomes of non-cardiogenic minor ischemic stroke patients with CMBs.

## Conclusion

The presence of CMBs was independently associated with a poor outcome in minor ischemic stroke patients.

## Data Availability Statement

All datasets generated for this study are included in the article/Supplementary Material.

## Ethics Statement

The studies involving human participants were reviewed and approved by the Ethics Committee of the Jikei University School of Medicine. The patients/participants provided their written informed consent to participate in this study.

## Author Contributions

KSaku designed the study, interpreted the data, and drafted the manuscript. TS designed the study and collected the data. TK designed the study and performed the statistical analysis. KSaka interpreted the data and drafted the manuscript. HM, SM, and HY designed the study and interpreted the data. YI designed the study, performed the statistical analysis, interpreted the data, and drafted the manuscript.

## Conflict of Interest

The authors declare that the research was conducted in the absence of any commercial or financial relationships that could be construed as a potential conflict of interest.

## References

[1] Cordonnier C Al-Shahi Salman R Wardlaw J. Spontaneous brain microbleeds: systematic review, subgroup analyses and standards for study design and reporting. Brain. (2007) 130:1988–2003. 10.1093/brain/awl387 17322562

[2] Greenberg SM Vernooij MW Cordonnier C Viswanathan A Al-Shahi Salman R Warach S. Cerebral microbleeds: a guide to detection and interpretation. Lancet Neurol. (2009) 8:165–74. 10.1016/S1474-4422(09)70013-4 19161908 PMC3414436

[3] Wilson D Charidimou A Ambler G Fox ZV Gregoire S Rayson P. Recurrent stroke risk and cerebral microbleed burden in ischemic stroke and TIA: A meta-analysis. Neurology. (2016) 87:1501–10. 10.1212/WNL.0000000000003183 27590288 PMC5075978

[4] Wang DN Hou XW Yang BW Lin Y Shi JP. Quantity of cerebral microbleeds, antiplatelet therapy, and intracerebral hemorrhage outcomes: a systematic review and meta-analysis. J Stroke Cerebrovasc. (2015) 24:2728–37. 10.1016/j.jstrokecerebrovasdis.2015.08.003 26342996

[5] Charidimou A Kakar P Fox Z Werring DJ. Cerebral microbleeds and recurrent stroke risk: systematic review and meta-analysis of prospective ischemic stroke and transient ischemic attack cohorts. Stroke. (2013) 44:995–1001. 10.1161/STROKEAHA.111.000038 23493732

[6] Boulanger JM Coutts SB Eliasziw M Gagnon AJ Simon JE Subramaniam S Cerebral microhemorrhages predict new disabling or fatal strokes in patients with acute ischemic stroke or transient ischemic attack. Stroke. (2006) 37:911–4. 10.1161/01.STR.0000204237.66466.5f 16469961

[7] Zand R Shahjouei S Tsivgoulis G Singh M McCormack M Noorbakhsh-Sabet N. Cerebral microbleeds are associated with higher mortality among ischemic stroke patients. J Stroke Cerebrovasc Dis. (2018) 27:3036–42. 10.1016/j.jstrokecerebrovasdis.2018.06.037 30093199

[8] Altmann-Schneider I Trompet S de Craen AJ van Es AC Jukema JW Stott DJ. Cerebral microbleeds are predictive of mortality in the elderly. Stroke. (2011) 42:638–44. 10.1161/STROKEAHA.110.595611 21233474

[9] Wilson D Werring DJ. Antithrombotic therapy in patients with cerebral microbleeds. Curr Opin Neurol. (2017) 30:38–47. 10.1097/WCO.0000000000000411 27898582

[10] Adams HP Jr Bendixen BH Kappelle LJ Biller J Love BB Gordon DL. Classification of subtype of acute ischemic stroke. Definitions for use in a multicenter clinical trial. TOAST. Trial of Org 10172 in Acute Stroke Treatment. Stroke. (1993) 24:35–41. 10.1161/01.STR.24.1.35 7678184

[11] Koennecke HC. Cerebral microbleeds on MRI: prevalence, associations, and potential clinical implications. Neurology. (2006) 66:165–71. 10.1212/01.wnl.0000194266.55694.1e 16434647

[12] Tsai HH Pasi M Tsai LK Chen YF Lee BC Tang SC. Microangiopathy underlying mixed-location intracerebral hemorrhages/microbleeds: a PiB-PET study. Neurology. (2019) 92:e774–81. 10.1212/WNL.0000000000006953 30674594 PMC6396971

[13] Fazekas F Chawluk JB Alavi A Hurtig HI Zimmerman RA MR signal abnormalities at 1.5 T in Alzheimer's dementia and normal aging. AJR Am J Roentgenol. (1987) 149:351–6. 10.2214/ajr.149.2.351 3496763

[14] Pan Y Jing J Li H Wang Y Wang Y He Y Abnormal glucose regulation increases stroke risk in minor ischemic stroke or TIA. Neurology. (2016) 87:1551–6. 10.1212/WNL.0000000000003200 27613582

[15] Jing J Meng X Zhao X Liu L Wang A Pan Y. Dual antiplatelet therapy in transient ischemic attack and minor stroke with different infarction patterns: subgroup analysis of the CHANCE randomized clinical trial. JAMA Neurol. (2018) 75:711–9. 10.1001/jamaneurol.2018.0247 29582084 PMC5885215

[16] Hankey GJ. Long-term outcome after ischaemic stroke/transient ischaemic attack. Cerebrovasc. Dis. (2003) 16(Suppl. 1):14–9. 10.1159/000069936 12698014

[17] Sobolewski P Brola W Wiszniewska M Szczuchniak W Fudala M Domagalski M. Intravenous thrombolysis with rt-PA for acute ischemic stroke within 24h of a transient ischemic attack. J Neurol Sci. (2014) 340:44–9. 10.1016/j.jns.2014.02.022 24635889

[18] Hoshino T Sissani L Labreuche J Bousser MG Chamorro A Fisher M. Non-cardioembolic stroke/transient ischaemic attack in Asians and non-Asians: a post-hoc analysis of the PERFORM study. Eur Stroke J. (2019) 4:65–74. 10.1177/2396987318797245 31165096 PMC6533862

[19] Johnston SC Easton JD Farrant M Barsan W Conwit RA Elm JJ. Clopidogrel and aspirin in acute ischemic stroke and high-risk TIA. N Engl J Med. (2018) 379:215–25. 10.1056/NEJMoa1800410 29766750 PMC6193486

[20] Pan Y Meng X Jing J Li H Zhao X Liu L. Association of multiple infarctions and ICAS with outcomes of minor stroke and TIA. Neurology. (2017) 88:1081–8. 10.1212/WNL.0000000000003719 28202699

[21] Kim JT Park MS Choi KH Kim BJ Han MK Park TH. Clinical outcomes of posterior versus anterior circulation infarction with low National Institutes of health stroke scale scores. Stroke. (2017) 48:55–62. 10.1161/STROKEAHA.116.013432 27856952

[22] Hankey GJ Secondary stroke prevention. Lancet Neurol. (2014) 13:178–94. 10.1016/S1474-4422(13)70255-2 24361114

[23] Lindley RI. Stroke prevention in the very elderly. Stroke. (2018) 49:796–802. 10.1161/STROKEAHA.117.017952 29371430

[24] Cucchiara B George DK Kasner SE Knutsson M Denison H Ladenvall P. Disability after minor stroke and TIA: a secondary analysis of the SOCRATES trial. Neurology. (2019) 93:e708–16. 10.1212/WNL.0000000000007936 31296654

[25] Georgakis MK Duering M Wardlaw JM Dichgans M. WMH and long-term outcomes in ischemic stroke: a systematic review and meta-analysis. Neurology. (2019) 92:e1298–308. 10.1212/WNL.0000000000007142 30770431

[26] Lovelock CE Cordonnier C Naka H Al-Shahi Salman R Sudlow CL Werring DJ. Antithrombotic drug use, cerebral microbleeds, and intracerebral hemorrhage: a systematic review of published and unpublished studies. Stroke. (2010) 41:1222–8. 10.1161/STROKEAHA.109.572594 20431083

[27] Cordonnier C. Brain microbleeds: more evidence, but still a clinical dilemma. Curr Opin Neurol. (2011) 24:69–74. 10.1097/WCO.0b013e328341f8c0 21124218

[28] Kim BJ Lee SH. Prognostic impact of cerebral small vessel disease on stroke outcome. J Stroke. (2015) 17:101–10. 10.5853/jos.2015.17.2.101 26060797 PMC4460329

[29] Rouhl RP Damoiseaux JG Lodder J Theunissen RO Knottnerus IL Staals J. Vascular inflammation in cerebral small vessel disease. Neurobiol Aging. (2012) 33:1800–6. 10.1016/j.neurobiolaging.2011.04.008 21601314

[30] Hainsworth AH Oommen AT Bridges LR. Endothelial cells and human cerebral small vessel disease. Brain Pathol. (2015) 25:44–50. 10.1111/bpa.12224 25521176 PMC8029339

[31] McAlpine H Churilov L Mitchell P Dowling R Teo S Yan B. Leukoaraiosis and early neurological recovery after intravenous thrombolysis. J Stroke Cerebrovasc. (2014) 23:2431–6. 10.1016/j.jstrokecerebrovasdis.2014.05.012 25174561

[32] Kissela B Lindsell CJ Kleindorfer D Alwell K Moomaw CJ Woo D. Clinical prediction of functional outcome after ischemic stroke: the surprising importance of periventricular white matter disease and race. Stroke. (2009) 40:530–6. 10.1161/STROKEAHA.108.521906 19109548 PMC2766300

[33] Leonards CO Ipsen N Malzahn U Fiebach JB Endres M Ebinger M. White matter lesion severity in mild acute ischemic stroke patients and functional outcome after 1 year. Stroke. (2012) 43:3046–51. 10.1161/STROKEAHA.111.646554 22935398

[34] Howard VJ Madsen TE Kleindorfer DO Judd SE Rhodes JD Soliman EZ. Sex and race differences in the association of incident ischemic stroke with risk factors. JAMA Neurol. (2019) 76:179–86. 30535250 10.1001/jamaneurol.2018.3862 PMC6439952

