# Supplementary material for: The Impact of Cerebral Microbleeds Presence on Outcome Following Minor Stroke Treated With Antiplatelet Therapy
Source: Front Neurol. 2020 Jun 16;11:522. doi: 10.3389/fneur.2020.00522 (PMC7308486; doi:10.3389/fneur.2020.00522)
Supplement: Supplementary file 1 [file Table_1.DOCX]

Supplementary method

Collecting clinical data

We collected the following clinical data from all patients: age, sex, time from symptom onset to admission, length of hospital stay, and traditional cardiovascular risk factors including: hypertension (defined as systolic blood pressure ≥ 140 mmHg or diastolic blood pressure ≥ 90 mmHg and/or medication with anti-hypertensive agents), dyslipidemia (defined as serum total cholesterol ≥ 5.69 mmol/L (220 mg/dL) or medication with anti-hyperlipidemic agents), diabetes mellitus (defined as medication with oral hypoglycemic agents or insulin, fasting plasma glucose ≥7.0 mmol/L (126 mg/dL), and glycosylated hemoglobin ≥ 6.5% according to the Japan Diabetes Society diagnostic criteria [11]); malignant neoplasms (defined as any lifetime experience of malignant neoplasms based on medical interviews); and chronic kidney disease (defined as an estimated glomerular filtration rate (eGFR) < 60 mL/min/1.73 m2 calculated from the serum creatinine level on admission (eGFR = 194 × (serum creatinine [mg/dL])−1.094 × age−0.287 × 0.739 [for women])). The National Institutes of Health Stroke Scale (NIHSS) was used on admission by a trained neurologist to quantify stroke severity.

Imaging Acquisition

All brain images were acquired using a 1.5-T scanner (MAGNETOM Avanto or MAGNETOM Symphony; Siemens Healthcare, Erlangen, Germany) or 3-T scanner (MAGNETOM Skyra; Siemens Healthcare, GmbH, Forchheim, Germany). The scan parameters for each sequence on the 1.5-T scanner were as follows: SWI (repetition time [TR] / echo time [TE] = 49/40ms; flip angle, 15 degrees; section thickness = 2 mm; field of view [FOV] = 23 cm; image matrix = 256 x 230 with interpolation), DWI (TR / TE = 2700/90 ms; section thickness = 5 mm; section gap = 1.5 mm; FOV = 21 cm; image matrix = 128 x 128 with interpolation), FLAIR (TR/TE = 9000/94 ms; flip angle, 160 degrees; section thickness = 5 mm; section gap = 1.5 mm; matrix = 256 x 256 with interpolation; FOV = 21 cm). The scan parameters for each sequence on the 3-T scanner were as follows: SWI (repetition time [TR] / echo time [TE] = 28/20 ms; flip angle, 15 degrees; section thickness = 1.5mm; field of view [FOV] = 240 mm; image matrix = 200 x 320 with interpolation), DWI (TR / TE = 5000/65 ms; section thickness = 4mm; section gap =1.6mm; FOV = 220 mm; image matrix= 160 x 160 with interpolation) , FLAIR (TR/TE = 9000/103 ms; flip angle, 170 degrees; section thickness = 4 mm; matrix = 192 x 384 with interpolation; FOV = 21 cm).
